# A Case Study Assessing the Auditory and Speech Development of Four Children Implanted with Cochlear Implants by the Chronological Age of 12 Months

**DOI:** 10.1155/2013/359218

**Published:** 2013-02-20

**Authors:** Birgit May-Mederake, Wafaa Shehata-Dieler

**Affiliations:** ^1^Cochlea Implantat Centrum (CIC) Süd, Berner Strasse 16, 97084 Würzburg, Germany; ^2^Audiology and Phoniatrics, Department of Otorhinolaryngology, Plastic, Aesthetic and Reconstructive Head and Neck Surgery, Comprehensive Hearing Center (CHC), University of Würzburg, Josef-Schneider Straße 11, 97080 Würzburg, Germany

## Abstract

Children with severe hearing loss most likely receive the greatest benefit from a cochlear implant (CI) when implanted at less than 2 years of age. Children with a hearing loss may also benefit greater from binaural sensory stimulation. Four children who received their first CI under 12 months of age were included in this study. Effects on auditory development were determined using the German LittlEARS Auditory Questionnaire, closed- and open-set monosyllabic word tests, aided free-field, the Mainzer and Göttinger speech discrimination tests, Monosyllabic-Trochee-Polysyllabic (MTP), and Listening Progress Profile (LiP). Speech production and grammar development were evaluated using a German language speech development test (SETK), reception of grammar test (TROG-D) and active vocabulary test (AWST-R). The data showed that children implanted under 12 months of age reached open-set monosyllabic word discrimination at an age of 24 months. LiP results improved over time, and children recognized 100% of words in the MTP test after 12 months. All children performed as well as or better than their hearing peers in speech production and grammar development. SETK showed that the speech development of these children was in general age appropriate. The data suggests that early hearing loss intervention benefits speech and language development and supports the trend towards early cochlear implantation. Furthermore, the data emphasizes the potential benefits associated with bilateral implantation.

## 1. Introduction

Best practice with regard to the timing of cochlear implantation continues to evolve as recent findings reveal convincing data related to the time of implantation and the achievement of maximum benefit for the young (<2 years old) [1–10]. The question of how early to intervene with cochlear implantation in children with a prelingual hearing loss is, therefore, currently one of the most clinically relevant topics since universal new-born hearing screening was introduced. To date several studies have shown that not only is surgery safe at a young age [1, 2], but also that the sooner implantation takes place, the greater the likelihood of a better outcome for the child's overall speech and language development [3–10].

It appears early implantation makes it possible for children with a hearing impairment to perform as well as their hearing peers in terms of speech and language development. Tomblin et al. [11] evaluated the expressive language growth of 29 infants and toddlers who received a CI and found that infants achieved a greater level of expressive language over a shorter period; achieving higher scores 3 years after implantation than their older counterparts. Similarly, Nicholas and Geers [12] compared the language skills of 76 children within the age range of 3.5 to 4.5 years who had been unilaterally implanted by the age of 36 months; they found that only those implanted before the age of 24 months had achieved age-appropriate spoken language levels by 4.5 years of age.

Further evidence indicates that a “sensitive” or “critical” period in speech and language development exists, during which implantation has the greatest potential impact [12–14]. The sensitive period of implantation appears to be limited to under 1 year of age [15], while the window of opportunity for language learning is determined from birth to 7 years of age [16]. Thus, early cochlear implantation in young children would extend the window of opportunity for language learning in hearing-impaired children by giving greater access to auditory stimuli, through spoken language across the window of opportunity. This serves to promote an increase in auditory skills, speech understanding, and oral linguistic development [17–19].

Unilateral amplification of bilateral hearing loss has been shown to be associated with limited auditory function [20–22]. The aim of binaural cochlear implantation is to improve speech recognition. When people with normal hearing listen with both ears, auditory function is improved [23]. Likewise, binaural CI use improves listening and speech recognition [24, 25] and limited evidence suggests that children in particular may benefit from binaural sensory stimulation (reviewed by Johnston et al. [26]). Furthermore, there is evidence that a sensitive age interval between implant surgeries may exist. A short interval between surgeries, of 6 to 12 months, probably enables children with a prelingual hearing loss to achieve good performance and the best opportunity for the subsequent development of speech and language skills [27, 28].

The purpose of this case study investigation was to describe the overall functioning over time of four children receiving bilateral cochlear implants at a young age (<2 years old). It was our aim to document and review the general auditory and speech behavior of these children. 

## 2. Materials and Methods

### 2.1. Subjects

Four children with a prelingual hearing loss (3 boys and 1 girl) were recruited from the Department of Otolaryngology and Cochlea Implantat Centrum (CIC) Süd, Würzburg, Germany, and enrolled in this longitudinal study. All children were being raised in a monolingual German speaking environment and no additional needs were reported. The etiology of congenital deafness of 3 children was unknown; one child (Case 3) had heredofamilial hearing loss. All the children had undergone cochlear implantation, before the chronological age of 12 months and were fitted with a MED-EL COMBI40+ and a behind the ear TEMPO+ speech processor. In all cases, the first fitting for both CIs took place within 1.5 months after implantation and no surgical complications were reported. The cognitive evaluation and speech and language assessment were undertaken in the rehabilitation center CIC Süd, where the children received auditory-verbal therapy in group and individual sessions. Audiological evaluations were at regular intervals in the Audiology Unit at the Department of Otolaryngology.

A parent or guardian gave written informed consent for each child at audiological assessment.

### 2.2. Assessment Procedure

#### 2.2.1. Cognitive Testing

For the purposes of this study it was important to rule out the presence of additional needs in overall intellectual functioning amongst children; thus, the data could be used comparatively to evaluate other children with the same degree of hearing loss and normal cognitive function. Nonverbal intelligence testing is a standard component of evaluations for patients seen at the CIC. Testing could only take place once the subjects had reached the lower age limits for which the tests were appropriate. In order to assess intellectual functioning with minimal interference of language skills, the children were tested with the SON-R 2.5-7 (German version, Snijers-Oomen Nonverbal Intelligence Test-Revision, 1980) for ages 2.5–7 [29]. The SON-R 2.5-7 uses a subset of tests for abstract thinking (categories and analogies), spatial thinking (mosaics and puzzles), and concrete thinking (situations and patterns). 

#### 2.2.2. Auditory Development

A complete battery assessing auditory behavior and speech development was performed at regular test intervals; beginning with unilateral testing and then bilateral once a second CI was fitted. The battery consisted of the LittlEARS Auditory Questionnaire [30], administered up to 24 months of hearing age; audiological evaluation at 3-4 month intervals, including aided free-field; and as soon as the child was able to, open-set speech audiometry. 

The aided free-field thresholds were measured using visual reinforcement (COR) and sound localization responses. Furthermore, the children were asked to localize a signal delivered at MCL through one of four loudspeakers arranged at 90°, 45°, −45°, and −90°. 

Speech audiometry was performed in a sound proof booth with the “Mainzer Kindersprachtest” [31] and the “Göttinger Kindersprachverständnistest” [32]. The Mainzer is a closed- or open-set German speech discrimination test. Participants must repeat words presented to them or identify the words by means of picture cards. Different test conditions are used for three age categories. The first test level is used for children under 4 years and is comprised of 10 words; the second level comprises 25 words for children aged 4-5 years; and the third test comprising 25 words is suitable for children aged 4-5 years. 

 The Göttinger is a German speech perception test for children aged 3-4 and 5-6 years. It comprises lists of words that the child being tested must repeat. Children can use picture cards to identify the words presented to them.

Reactions to sounds and speech perception abilities were tested with the Listening in Progress Profile (LiP), the Monosyllabic-Trochee-Polysyllabic (MTP) test, and closed- and open-set monosyllabic word tests. These tests are part of the evaluation of auditory responses to speech (EARS) test battery [30]. 

The LiP was used to assess the subjects' auditory detection, discrimination, and early identification abilities of environmental sounds and speech (at the single word or phoneme level); LiP scores were reported as percentage correct. The MTP test is a closed-set test used to assess the ability of an individual in recognizing words with different syllabic patterns. These tests were performed in a quiet room under normal ambient noise conditions.

#### 2.2.3. Speech Production and Grammar Development

To investigate the speech development of the children in the present case series the “Sprachentwicklungstest” (SETK) was used [33, 34]. The SETK is an age-specific test for children between 2 and 5 years of chronological age. The SETK-2 test, for 2-year-old children, is comprised of 4 subtests: Word Comprehension, Sentence Comprehension, Word Production and Sentence Production. The SETK-3–5 for children aged 3–5 years comprises 4 subtests for children up to 4;11 years and the SETK-4-5 comprises 5 subtests for children aged 4-5;11 years old. Children up to 4;11 years are tested in Sentence Comprehension, the Encoding of Semantic Relations, Morphological Syntax, and Phonological Working Memory for Nonsense Words. Likewise, children aged 4-5;11 are tested in Sentence Comprehension, Morphological Syntax, and Phonological Working Memory for Nonsense Words and, in addition, Speech Repetition and the Memory Span of words. 

The TROG-D is a German adapted test which aims to determine the reception of grammar in children aged 3–10;11 years [35]. The TROG-D assesses grammatical comprehension by measuring the understanding of 18 different sentence constructions. Each sentence construction is presented 4 times each using different test stimuli. 

The AWST-R [36], “Aktiver Wortschatztest” or active vocabulary test is a test for German speaking children between 3 and 5.5 years old. It is a test of vocabulary expression in which participants verbalize one-word responses to pictures shown. The test consists of 51 nouns and 24 verbs presented graphically. The child's response in word expression is appraised by a qualitative score. 

### 2.3. Statistical Analyses

The interpretation of referenced test results was performed via comparison to a normative group of hearing children. The values of the normative group are represented as T-values and percentage range for a given test, which is determined and provided by the test developer. The T-values of the CI children were compared to the mean normative T-values of the hearing children. Results of TROG-D, AWST-R, SETK-2, SETK-3–5, and SETK-4-5 are depicted in a table format.

## 3. Results

### 3.1. Cognitive Testing

Except for the severe hearing loss the subjects were neurologically and intellectually age appropriate as determined by the SON-R 2.5–7 nonverbal intelligence test (see Table 1 in Supplementary Material available online at http://dx.doi.org/10.1155/2013/359218).

### 3.2. Case 1

#### 3.2.1. Case History

The female subject was implanted at the age of 9;24 months, and the first fitting was performed at the age of 11;26 months of age in the left ear, following 1-month hearing aid experience with no listening progress. The child had no preoperative speech perception skills at the beginning of this study. Initial fitting on the right ear called for louder levels until the maximum was reached and the need arose for the fitting of a second CI at 18 months of age in the left ear. At this point, stimulation on the right side was reduced and fitting levels remained stable. 

#### 3.2.2. Auditory Development

**Figure 1:**
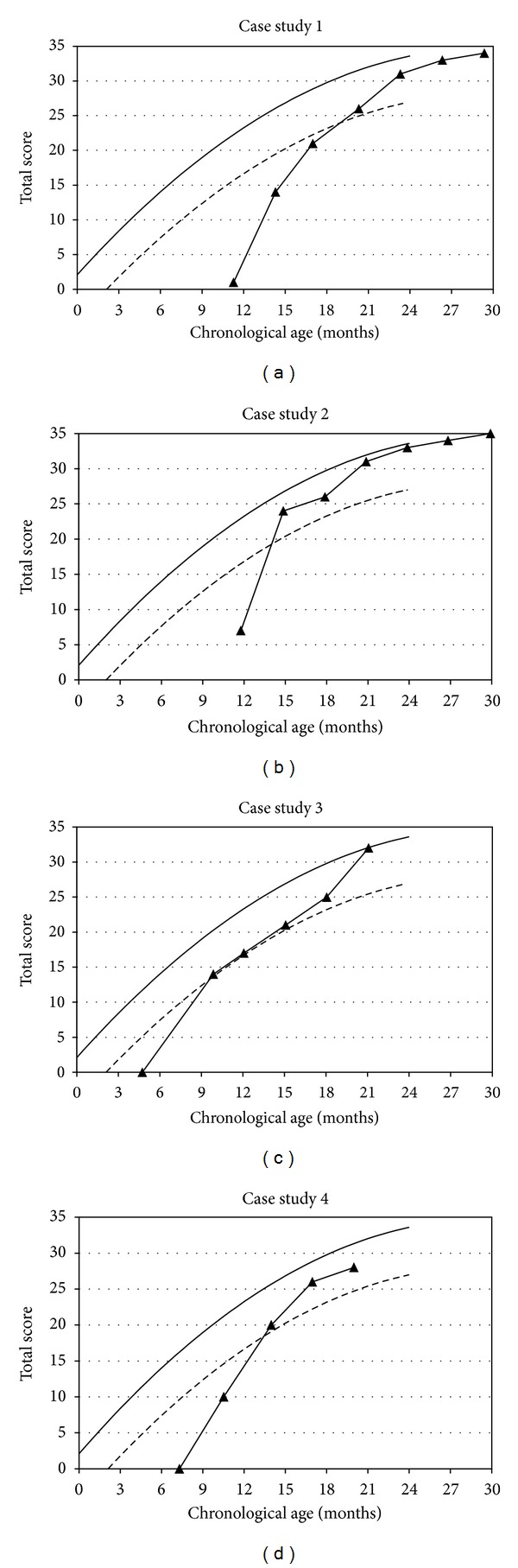
Auditory development as determined by the LittlEARS Auditory Questionnaire ((a)–(d)). The solid line denotes the German derived normative curve. The dashed line represents the minimum 95% confidence interval values from the German-derived norms. Filled triangles denote the score of children (*y*-axis) at chronological age (*x*-axis).

Auditory development as determined by the LittlEARS Auditory Questionnaire improved from a score of 0 preoperatively to 1 at the first interval to a score of 14, 21, 26, and 31 at subsequent intervals (Figure 1(a)). The child's rapid growth in auditory skills at 20 months of age put her within the expected level of achievement for hearing children. Following bilateral implantation, the scores were 100% after the 15th and 21st months.

Prior to implantation the child's aided responses with the hearing aids were at 0.5−1 kHz 70 dB with a steep drop at 2–4 kHz to 90 dB. Four months after the first fitting of the implanted side aided responses with the CI were at 0.5–4 kHz 40–45 dB. Similarly, aided responses in free-field showed continuous improvement. Ten months after the first fitting of the first CI and 2 months after the fitting of the second CI the child reacted at 0.5–4 kHz at 25–35 dB. 

Sound localization abilities also showed continuous improvement following the fitting of the second CI. After 6 months of experience with the first CI the child was unable to localize sounds correctly, from 90 and −90 loudspeakers. Two months after the fitting of the second implant the child was able to localize 100% of the signals offered from all 4 loudspeakers. This was consistent after 6 and 12 months of binaural hearing experience.

Twenty-two months after the first fitting of the first CI the child was able to reach an 80% word discrimination score at 70 dB on the Mainzer Test (mono- and bisyllabic words). At a hearing age of 30 months the child's word discrimination score was 90% at 70 dB on the Göttinger test (monosyllabic words). 

**Figure 2:**
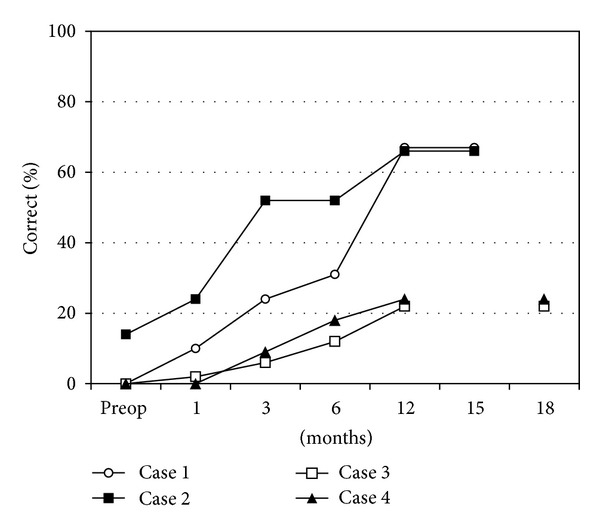
Listening Progress Profile (LiP) at follow-up, preoperatively (preop), at 1-month and at 3-month intervals. LiP scores are reported as percentage correct.

At follow-up LiP percentages correct were 10% at first fitting, 23% after 3 months, 31% after 6 months, 66% after 12 months, and 66% after 15 months CI experience (Figure 2).

MTP testing at the 12-word level was 100% at the 18-month interval. At 24 months the child had reached 100% in the monosyllable test (closed-set) and 50% discrimination for monosyllables in the open-set test. At 31 months she reached a score of 100% in the open-set monosyllables test. 

#### 3.2.3. Speech Production and Grammar Development

At the age of 2;11 years SETK-2 showed that the child's “Word Comprehension” “Sentence Comprehension”, and “Word Production” were within the normative range of hearing children (Table 1). However, the child's “Sentence Production” was below the normative range of hearing children. 

At the age of 3;11 years SETK-3–5 testing indicated that the child's “Sentence Comprehension”, “Encoding of Semantic Relations”, and “Morphological Syntax” were within the range of the normative hearing group (Table 1). The T-value of “Phonological Working Memory for Nonsense Words” was slightly below the range of hearing children.

With 4;7 years of chronological age the SETK-4-5 was conducted (Table 1). The results in the subtests “Sentence Comprehension” and “Phonological working memory for Nonsense Words” were within the range of the hearing children. The “Morphological Syntax” and “Sentence Memory” of the cochlear implanted child were slightly below the normative range. Likewise, the “Memory Span” of word orders, with 3 words correct, was slightly below average. 

In the TROG-D test, conducted at 4;10 years of age the child reached a T-value which was representative of the normative range of hearing children (Table 2). 

In the vocabulary test AWST-R, conducted at the age of 4;8, years the child's performance was within the normative range of hearing children (Table 2). 

### 3.3. Case 2

#### 3.3.1. Case History

The male subject presented was implanted at the age of 10;10 months on the right side and 19;1 months on the left side. Prior to fitting the child had no hearing experience, despite the use of hearing aids on both sides since the age of three months. Within days of the first fitting the right CI was increased by 30% of the original stimulation levels. After fitting the left CI (at approximately the 8-month interval) the stimulation levels, on the right CI, were reduced to the original fitting parameters. 

#### 3.3.2. Auditory Development

Out of 35 questions on the LittlEARS Auditory Questionnaire results improved from 7 at the first interval to 24, 26, and 31 at consecutive intervals. As shown in Figure 1(b), at the 14-month interval the child had reached a level within the range of hearing children, illustrated by the lower limits of expected functioning for his age (95% confidence interval).

Prior to implantation the child's aided responses with the hearing aids were at 0.5 kHz 85 dB, at 1 kHz 80 dB, and at 2 kHz 90 dB. Three months after fitting of the first implant the child's aided responses with the CI were at 0.5–4 kHz 30 dB. Aided responses remained stable throughout the follow-up period. 

The child's sound localization abilities showed continuous improvement following the fitting of the second CI. Four months after the fitting the second implant the child was able to localize 75% of the signals offered correctly, from all 4 loudspeakers. This was consistent after 6 and 9 months of binaural hearing experience.

Thirteen months after the first fitting of the first CI the child reached 80% word discrimination scores at 70 dB on the Mainzer Test (mono-and bisyllabic words). At a hearing age of 20 months his word discrimination score at 70 dB on the Göttinger test (monosyllabic words) was 80%. 

Likewise, listening and perception of speech and environmental sounds improved as determined by LiP test score. Compared to 14% preoperatively the LiP score was 24% at the first fitting, 52% at 3 months, 52% at 6 months, and 66% after 12 months (Figure 2). 

MTP test results identifying speech patterns at the 12-month interval indicated 100% of both the 3-word and the 3-pattern subtests were correct and 84% on the 6-word subtest and 89% on the 6-pattern subtest. At the 15-month interval MTP performance in the 6-word and 6-pattern subtests increased to 100% and testing of both the 12-word and pattern level were 67% correct. At the 22-month interval, 100% of the words on the 12-word subtest, which is the most advanced level of difficulty for the MTP test, were correct. This was an improvement from 67% at the 15-month interval on the same test. At 24 months the child has reached 100% on the monosyllable test (closed-set) and 70% discrimination on the open-set monosyllables test. At 28 months he reached 100% on the open-set monosyllables test.

#### 3.3.3. Speech Production and Grammar Development

At the age of 2;3 years the SETK-2 was conducted (Table 1). The “Word Comprehension” test result was within the range of hearing children. The child's performance in “Word Production” was higher than average, but the “Sentence Comprehension” and “Sentence Production” subtests were not performed on account of the child's lack of cooperation. 

At 3;11 years the SETK-3–5 was conducted (Table 1). The child refused to cooperate upon testing of the “Encoding of Semantic Relations”. The child's “Sentence Comprehension” and “Phonological Working Memory for Nonsense Words” (T-value: 57; 75.8%) were within the normative range of hearing children. The child's performance of “Morphological Syntax” was greater than the average hearing child's.

At the age of 4;10 years the SETK-4-5 (Table 1) indicated that the subject's “Sentence Comprehension” (T-value: 63; 90.3%) and “Morphological Syntax” (T-value: 63; 90.3%) were greater than the normative group of hearing children. Similarly, in comparison to the normative hearing children group his performance on “Phonological Working Memory for Nonsense Words” and “Speech Repetition” was greater. The child's “Memory Span,” with 4 words, was within the range of the normative group. 

At 5;2 years the TROG-D, used to determine the child's understanding of grammar, was within the normative range of hearing children (Table 2).

Likewise, at the same age the vocabulary as determined by AWST-R indicated that the child was within the normative range of his hearing peers (Table 2).

### 3.4. Case 3

#### 3.4.1. Case History

The male subject presented was fitted with hearing aids at 3 months of age with no apparent benefit. He was first implanted in the right ear at 4;21 months with a CI and then contralaterally at 16 months of age. 

#### 3.4.2. Auditory Development

In the LittlEARS Auditory Questionnaire the child achieved a score of 0 at the first interval and a score of 14, 17, 21, 25, 32, 33, 32, and 34 at subsequent intervals (Figure 1(c)). The LittlEARS Auditory Questionnaire scores were within the lower 95% confidence interval of expected values at the second test interval and remained at or above this benchmark at subsequent testing.

Prior to implantation the child showed minimal response with a hearing aid. In free-field the child's reactions with both hearing aids were inconsistent at 0.5, 1, and 2 kHz at 100–120 dB. Three months after fitting the first implant the child's aided responses with the CI were at 0.5 to 4 kHz 65–80 dB. His aided responses in free-field continued to show an improvement. Six months after the first fitting of the first CI he reacted at 45–55 dB. Eighteen months after the first fitting of his first CI and 6 months after the fitting of his second CI the child reacted at 35–40 dB. The child's aided thresholds remained stable throughout the rest of the follow-up period. 

The child's sound localization abilities showed a continuous improvement following the fitting of the second CI. Six months after the fitting the child was able to localize 100% of the signals offered from all 4 loudspeakers. This was consistent after 9 and 18 months of binaural hearing experience.

Twenty-eight months after the first fitting of the first CI the child reached an 80% word discrimination scores at 70 dB on the Mainzer Test (mono-and bisyllabic words). 

The scores of the LiP profile were as follows: 0% preoperatively, 4% at the 1-month interval, 6% at 3 months, 28% at 6 months, 52% at 12 months, and 62% at the 18-month interval (Figure 2).

The score reached on the MTP test was 100%, on the 3-word and 6-word tests, at the 12- and 18-month intervals. The child scored 100% on the MTP 12-word test at the 24-month interval. At the same interval (24 months) the child scored 80% on the open-set monosyllable test. At the 28-month interval the child reached a score of 100% on the closed- and open-set monosyllable tests.

#### 3.4.3. Speech Production and Grammar Development

When the child was 2;10 years old the SETK-2 was conducted (Table 1). The subject refused to cooperate for the “Sentence Production” subtest. The results of the remaining subtests “Word Comprehension,” “Sentence Comprehension”, and “Word Production” were within or greater than the normative range of hearing children.

At the chronological age of 3;4 years the “Sentence Comprehension” of the child determined by SETK-3–5 (Table 1) indicated the child's performance was within the range of the normative group of hearing children. Likewise, at 3;6 years the “Encoding of Semantic Relations” was within the normative range of hearing children. The remaining subtests were not performed due to organizational reasons.

At 4;0 years of age the SETK 4-5 was conducted (Table 1) and the child scored within the normative range of hearing children for each of the following subtests: “Morphological Syntax,” “Sentence Comprehension”, and “Sentence Memory.” The subtest “Phonological Working Memory for Nonsense Words” was not finished, due to lack of cooperation on the child's part. The “Memory Span” for word order was 4, which was within the normative range of hearing children.

At the chronological age of 4;3 years the grammatical test TROG-D was conducted. The child's performance was within the normative range of hearing children (Table 2). 

The vocabulary test AWST-R conducted at an age of 3;8 showed the child's performance was higher than the average of the normative hearing children group (Table 2).

### 3.5. Case 4

#### 3.5.1. Case History

The male subject was fitted with hearing aids at the age of 4 months. Following the hearing aid trial, the child received his first CI on the right side at the age of 6;15 months and experienced the need for louder and louder settings at fitting, until a contralateral CI was fitted shortly after the 12-month interval. Stimulation rates on the right side were subsequently decreased by 10%. No alterations to the initial fitting parameters of the second CI, on the left side, were made. 

#### 3.5.2. Auditory Development

The LittlEARS Auditory Questionnaire determined that auditory performance, which was 0 preoperatively, improved from a score of 10 at the first interval to scores of 20, 26, 30, 35, 34, and 35 at subsequent intervals (Figure 1(d)). Scores on the LittlEARS Auditory Questionnaire reached the lower 95% confidence interval for the normal range of hearing children.

Prior to implantation the child showed hardly any response with the hearing aid. In free-field the child did not show any consistent reactions to sounds presented at maximum intensity (120 dB). Three months after fitting the first implant the child's aided responses with the CI were at 0.5–4 kHz 55–70 dB. The child's aided responses in free-field slowly, but continuously, improved over time. Six months after the first fitting of the first CI the child reacted at 50–80 dB. Twenty-four months after the first fitting of the first CI and 15 months after the fitting of the second CI the child reacted at 0.5 to 8 kHz at 30–35 dB. The aided thresholds remained stable throughout the rest of the follow-up period. 

The child's sound localization abilities showed continuous improvement following the fitting of the second CI. After five months the child was able to localize 90% of the signals offered from all 4 loudspeakers. At 8 and 12 months of binaural hearing experience the child was able to localize 100% of the sound sources. 

Twenty-one months after the first fitting of the first CI the child reached 90% word discrimination at 70 dB on the Mainzer Test (mono- and bisyllabic words). At a hearing age of 24 months discrimination scores on the Göttinger Test (monosyllabic words) were 50%.

Scores on LiP testing were 0% preoperatively and at the 1-month interval; 21% at the 3-month interval; 42% at the 6-month interval; and 57% at the 12-month interval (Figure 2). 

The MTP 12-word score obtained at the 18-month interval was 100%. At 18 months the child also reached 100% on the monosyllable test (closed-set). At a hearing age of 24 months, discrimination of open-set monosyllables was 80%. 

#### 3.5.3. Speech Production and Grammar Development

At the age of 2;5 years the single subtests of SETK-2 were conducted (Table 1). The child's “Word Comprehension” and “Sentence Comprehension” were both within the normative range of hearing children. Determination of the child's “Word Production” and “Sentence Production” were not performed because the child refused to cooperate.

At 4;4 years of age the SETK 4-5 determined that the child had greater than average performance in the “Morphological Syntax” subtest compared to the normative range of hearing children (Table 1). Similarly, the child performance in “Sentence Comprehension”, “Sentence Memory”, and “Memory Span” of 4-word orders were within the normative range. The “Phonological Working Memory for Nonsense Words” subtest was not conducted on account of the child's difficulty with articulation. 

At 4;8 years of age the child's understanding of grammar tested via TROG-D revealed that his performance was within the normative range of hearing children (Table 2). 

Similarly, according to AWST-R the subject's vocabulary at 4;6 years was within range when compared to the normative group (Table 2). 

## 4. Discussion

Cochlear implantation in children under 2 years of age aims to expose children with a hearing loss to spoken language via hearing, thus, minimizing the gap between chronological age and the development of language skills that may occur in children with a hearing loss compared to their hearing peers [16, 17]. The data presented herein, of four CI recipients with normal cognitive development, implanted binaurally at less than 18 months of chronological age, indicates that early implantation supports age-appropriate auditory skills. Furthermore, audiological findings improved over time according to closed-set and open-set monosyllabic word test results, aided free-field, speech audiometry, LiP and MTP results. Localization abilities improved as early as 2 months after binaural hearing and continued to improve over time. Age-appropriate SETK testing illustrated that the speech development of these children was in general within the normative range of hearing children. A specific improvement in the grammar and vocabulary development of these children was also observed after receiving a CI compared to the normative range of hearing children and over time compared to their own performance at first fitting. 

Growth curves of the children, reflected in the results of the LittlEARS battery, show individual differences in each child's progress and rapid development. All achieved age-appropriate auditory skills compared to the normative range of hearing children, as determined by the LittlEARS Auditory Questionnaire, by 20 months of age (range = 10–20 months). This supports the findings of Tomblin et al. [11] and Nicholas and Geers [12] that show that closing the gap in development is not only possible, but also probable for very young CI users. Moreover, the present study illustrates that similar benefits can be achieved through bilateral implantation of young children. Although the current data did not specifically look at the effects of unilateral versus bilateral CI use on speech and language development, it illustrates that the benefits achieved through unilateral CI use also apply to bilaterally implanted children. Furthermore, the data supports the current trend toward decreasing the chronological age of cochlear implantation in children with a hearing loss [37].

Early exposure to auditory stimuli via cochlear implantation under 2 years affords children with greater access to spoken language via hearing, which promotes a subsequent increase in auditory and linguistic skills and speech understanding [17–19]. The capacity of the child to achieve these skills is likely to develop due to extensive neuroplasticity in children. The functional development of stimulus-driven complex neural processes and their role in the networks of the auditory system underpin early auditory stimuli [38]. A basic principle of developmental biology is that certain areas of the cortex hold the potential to reorganize if appropriate stimulation is withheld for long enough periods. Furthermore, it appears that there are critical or sensitive periods of neurobiological development in the brain during which behavioural responses can be learned (reviewed by Bischof et al. [38, 39]). In the auditory system, the sensitive period is during the time the brain is “maximally plastic” and “primed for stimulation-driven development” [40]. Thus, we assume that when stimulus is presented within the sensitive period, despite the lack of initial stimuli (as in the case of children with a prelingual hearing loss), the child can subsequently achieve age-appropriate speech and language skills. As neuroplasticity declines with increasing age, it is reasonable to assume the child's capacity to achieve these skills diminishes. Sharma et al. indicate that the sensitive period of plasticity in children with a CI lasts up to 3.5 years of age [14, 40]. Less specifically numerous studies show the beneficial effects in children, on speech and language development, of a CI when implanted before 2 years of chronological age [7, 41].

The data described in the present study is suggestive of a sensitive period. All children were bilaterally implanted before 2 years of age and showed “Word Comprehension” and “Sentence Comprehension” abilities within the normative range of hearing children when followed up by SETK-2. Likewise, “Word Production” tested by SETK-2 was within the range of hearing children. By the time of SETK-4-5 all the children's performance in “Sentence Comprehension” were within, or above, the normative range of hearing children and the same applied for “Speech Repetition” in three out of four of the children. The fourth child's “Speech Repetition” fell only marginally below that of the normative group. Similarly, Vlastarakos et al. has shown that age at implantation shows the greatest influence on speech perception, speech production, and language outcomes in children implanted with a CI between 1 to 72 months of age [37]. Age at fitting accounted for the largest variance in both speech perception and speech and language production [37]. Missed tests and the refusal of children to test may contribute to the variance observed. Likewise, we suggest that this is an important determinant of the outcomes of the parameters we have looked at. 

Given the evidence indicating that the development of language skills, in children with a prelingual hearing loss, requires several years to follow-up [42], a follow-up period of up to 5 years of chronological age in the present study is an appropriate indicator of individual performance. The most prominent effects over time were observed using SETK. All children receiving an implant in this study tested with the SETK-2, -3–5, or -4-5 performed within the range of hearing children. This indicates that, despite the gap in hearing versus chronological age, over time children with a CI are able to attain age-equivalent language skills, narrowing, or eliminating, the potential gap between them and hearing children in speech development. Furthermore, the children having received their first CI before 12 months of age, and followed up using AWST-R between 3 and 5.5 years and TROG-D between 3 and 11 years, were all within the normative range of hearing children. One child in particular, as determined by qualitative score in these tests performed better than the normative group of hearing children in terms of word expression. 

The data presented in this series illustrates each individual child's educational and social needs. This emphasizes the need to consider the appropriateness of habilitation for each child on an individual basis. We suggest that, according to the results herein, cochlear implantation with a CI at less than 2 years of age may be of significant benefit to children with a hearing loss. It appears that the children gain in auditory, speech, and grammar development upon the use of a CI. The potential benefits support the trend toward early cochlear implantation. Furthermore, as the children included in this study were all bilateral CI recipients, the data emphasizes the potential benefits associated with bilateral implantation. Although we did not specifically address differences in unilateral versus bilateral implantation, we can conclude that the bilateral implantation has contributed to the capacity of the children to develop age-appropriate auditory speech and language skills. 

## Supplementary Material

Results of German Snijers-Oomen Nonverbal Intelligence Test.

## Figures and Tables

**Table 1:** Outcomes of age-appropriate speech development tests SETK-2, SETK-3–5, and SETK-4-5, presenting T-values of four bilaterally cochlear implanted children, implanted at under two years of age.

	Case 1	Case 2	Case 3	Case 4
	T-value (%)	T-value (%)	T-value (%)	T-value (%)
SETK-2				

Comprehension I and II:				
Word comprehension	45 (30.8)	54 (65.5)	61 (88.5)	54 (65.5)
Sentence comprehension	41 (18.4)	—	41 (18.4)	54 (65.5)
Speech production I and II:				
Word production	47 (38.2)	76 (99.5)	56 (72.6)	—
Sentence production	34 (5.5)	—	—	—

SETK-3–5				

Sentence comprehension	50 (50.0)	55 (69.2)	50 (50.0)	—
Encoding semantic relations	45 (30.9)	—	65 (93.3)	—
Morphological syntax	42 (21.2)	62 (88.5)	—	—
Phonological working memory for nonsense words	39 (13.6)	57 (75.8)	—	—

SETK-4-5				

Sentence comprehension	55 (69.2)	62 (88.5)	53 (61.8)	47 (38.2)
Morphological syntax	39 (13.6)	63 (90.3)	41 (18.4)	64 (91.9)
Phonological working memory for nonsense words	46 (34.5)	43 (24.2)	—	—
Memory span (word)	38 (11.5)	58 (78.8)	49 (46.0)	55 (69.2)

—indicates child was not tested or refused cooperation at this test interval.

% indicates percentile rank.

**Table 2:** Outcomes (T-values (Percentile rank)) of: (a) test for the reception of grammar (TROG-D) and; (b) active vocabulary test (AWST-R), in 4 (Case 1–4) children with bilateral cochlear implants.

	Case 1	Case 2	Case 3	Case 4
	T-value (%)	T-value (%)	T-value (%)	T-value (%)
(a) Reception of grammar: TROG-D	54 (64.0)	54 (65.0)	54 (64.0)	57 (77.0)
(b) Active vocabulary: AWST-R	49 (48.0)	56 (73.0)	50 (95.0)	42 (45.0)

## References

[1] Black IM, Bailey CM, Albert DM (2007). The Great Ormond Street Hospital paediatric cochlear implant programme 1992–2004: a review of surgical complications. *Cochlear Implants International*.

[2] Colletti L (2009). Long-term follow-up of infants (411 months) fitted with cochlear implants. *Acta Oto-Laryngologica*.

[3] Ertmer DJ, Young NM, Nathani S (2007). Profiles of vocal development in young cochlear implant recipients. *Journal of Speech, Language, and Hearing Research*.

[4] Nicholas JG, Geers AE (2006). Effects of early auditory experience on the spoken language of deaf children at 3 years of age. *Ear and Hearing*.

[5] Watson LM, Archnold SM, Nikolopoulos TP (2006). Children’s communication mode five years after cochlear implantation: changes over time according to age at implant. *Cochlear Implants International*.

[6] Anderson I, Weichbold V, D’Haese PSC (2004). Cochlear implantation in children under the age of two—what do the outcomes show us?. *International Journal of Pediatric Otorhinolaryngology*.

[7] Manrique M, Cervera-Paz FJ, Huarte A, Molina M (2004). Advantages of cochlear implantation in prelingual deaf children before 2 years of age when compared with later implantation. *Laryngoscope*.

[8] Richter B, Eißele S, Laszig R, Löhle E (2002). Receptive and expressive language skills of 106 children with a minimum of 2 years’ experience in hearing with a cochlear implant. *International Journal of Pediatric Otorhinolaryngology*.

[9] Hammes DM, Novak MA, Rotz LA, Willis M, Edmondson DM, Thomas JF (2002). Early identification and cochlear implantation: critical factors for spoken language development. *The Annals of Otology, Rhinology and Laryngology*.

[10] Kirk KI, Miyamoto RT, Lento CL, Ying E, O’Neill T, Fears B (2002). Effects of age at implantation in young children. *The Annals of Otology, Rhinology and Laryngology*.

[11] Tomblin JB, Barker BA, Spencer LJ, Zhang X, Gantz BJ (2005). The effect of age at cochlear implant initial stimulation on expressive language growth in infants and toddlers. *Journal of Speech, Language, and Hearing Research*.

[12] Nicholas JG, Geers AE (2007). Will they catch up? The role of age at cochlear implantation in the spoken language development of children with severe to profound hearing loss. *Journal of Speech, Language, and Hearing Research*.

[13] Svirsky MA, Teoh SW, Neuburger H (2004). Development of language and speech perception in congenitally, profoundly deaf children as a function of age at cochlear implantation. *Audiology and Neuro-Otology*.

[14] Sharma A, Dorman MF, Spahr AJ (2002). A sensitive period for the development of the central auditory system in children with cochlear implants: implications for age of implantation. *Ear and Hearing*.

[15] Dettman SJ, Pinder D, Briggs RJS, Dowell RC, Leigh JR (2007). Communication development in children who receive the cochlear implant younger than 12 months: risks versus benefits. *Ear and Hearing*.

[16] Waltzman SB, Roland JT (2005). Cochlear implantation in children younger than 12 months. *Pediatrics*.

[17] Houston DM, Pisoni DB, Kirk KI, Ying EA, Miyamoto RT (2003). Speech perception skills of deaf infants following cochlear implantation: a first report. *International Journal of Pediatric Otorhinolaryngology*.

[18] Robbins AM, Osberger MJ, Miyamoto RT, Kessler KS (1995). Language development in young children with cochlear implants. *Advances in Oto-Rhino-Laryngology*.

[19] Al-Muhaimeed H (2010). Assessment of auditory performance in young children with cochlear implants. *Cochlear Implants International*.

[20] Gatehouse S (1992). The time course and magnitude of perceptual acclimatization to frequency responses: evidence from monaural fitting of hearing aids. *Journal of the Acoustical Society of America*.

[21] Gelfand SA, Silman S (1993). Apparent auditory deprivation in children: implications of monaural versus binaural amplification. *Journal of the American Academy of Audiology*.

[22] Hattori H (1993). Ear dominance for nonsense-syllable recognition ability in sensorineural hearing-impaired children: monaural versus binaural amplification. *Journal of the American Academy of Audiology*.

[23] Ricketts T, Lindley G, Henry P (2001). Impact of compression and hearing aid style on directional hearing aid benefit and performance. *Ear and Hearing*.

[24] Boggess W, Balkany T, Dinner B (1988). Binaural cochlear implantation: comparison of 3 M/House and Nucleus 22 devices with evidence of sensory integration. *Laryngoscope*.

[25] Pijl S (1991). Single-channel versus bilateral multichannel cochlear implant results: a case report. *Ear and Hearing*.

[26] Johnston JC, Durieux-Smith A, Angus D, OConnor A, Fitzpatrick E (2009). Bilateral paediatric cochlear implants. A critical review. *International Journal of Audiology*.

[27] Papsin BC, Gordon KA (2008). Bilateral cochlear implants should be the standard for children with bilateral sensorineural deafness. *Current Opinion in Otolaryngology and Head and Neck Surgery*.

[28] Kühn-Inacker H, Shehata-Dieler W, Müller J, Helms J (2004). Bilateral cochlear implants: a way to optimize auditory perception abilities in deaf children?. *International Journal of Pediatric Otorhinolaryngology*.

[29] Tellegen PJ, Laros JA, Petermann F (2007). *SON-R 2 1/2−7 Non-Verbaler Intelligenztest*.

[30] Coninx F, Weichbold V, Tsiakpini L (2003). *LittlEARS Auditory Questionnaire*.

[31] Grimm H, Aktas M, Frevert S (2000). *Sprachentwicklungstest für zweijährige Kinder*.

[32] Grimm H (2001). *SETK 3–5. Sprachentwicklungstest für drei—bis fünfjährige Kinder. Diagnose von Sprachverarbeitungsfähigkeiten und auditiven Gedächtnisleistungen*.

[33] Biesalski P, Leitner H, Leitner E, Gaugel P (1974). Der Mainzer Kindersprachtest. Sprachaudiometrie im Vorschulalter. *HNO*.

[34] Gabriel P, Chilla R, Kiese C, Kabas M, Bänsch D (1976). Der Göttinger Kindersprachverständnistest II. *HNO*.

[35] Kampfhaus RW (2005). *Clinical Assessment of Child and Adolescent Intelligence*.

[36] Kiese-Himmel C (2005). *Aktiver Wortschatztest für 3-bis 5-jährige Kinder*.

[37] Vlastarakos PV, Proikas K, Papacharalampous G, Exadaktylou I, Mochloulis G, Nikolopoulos TP (2010). Cochlear implantation under the first year of age-The outcomes. A critical systematic review and meta-analysis. *International Journal of Pediatric Otorhinolaryngology*.

[38] Sininger YS, Grimes A, Christensen E (2010). Auditory development in early amplified children: factors influencing auditory-based communication outcomes in children with hearing loss. *Ear and Hearing*.

[39] Bischof HJ (2007). Behavioral and neuronal aspects of developmental sensitive periods. *NeuroReport*.

[40] Sharma A, Nash AA, Dorman M (2009). Cortical development, plasticity and re-organization in children with cochlear implants. *Journal of Communication Disorders*.

[41] Sharma A, Dorman MF, Kral A (2005). The influence of a sensitive period on central auditory development in children with unilateral and bilateral cochlear implants. *Hearing Research*.

[42] Waltzman SB, Cohen NL (1998). Cochlear implantation in children younger than 2 years old. *American Journal of Otology*.

