# Supplementary material for: A Case Study Assessing the Auditory and Speech Development of Four Children Implanted with Cochlear Implants by the Chronological Age of 12 Months
Source: Case Rep Otolaryngol. 2013 Feb 20;2013:359218. doi: 10.1155/2013/359218 (PMC3590554; doi:10.1155/2013/359218)
Supplement: Supplementary file 1 — Results of German Snijers-Oomen Nonverbal Intelligence Test. [file 359218.f1.docx]

**Supplemental Table 1:** Results of German Snijers-Oomen Nonverbal Intelligence Test.

| **CATEGORY** | **CASE 1** | **CASE 2** | **CASE 3** | **CASE 4** |
| --- | --- | --- | --- | --- |
| **Mosaics** | 7 | 12 | 12 | 14 |
| **Categories** | 13 | 14 | 6 | 11 |
| **Puzzles** | 7 | 17 | 11 | 13 |
| **Analogies** | 11 | 16 | 8 | 13 |
| **Situations** | 9 | 14 | 10 | 9 |
| **Patterns** | 9 | 8 | 13 | 12 |
| **Total IQ (Percentile Rank)** | 95 (37) | 126 (96) | 100 (50) | 115 (84) |
